# Exosomal Interventions in Bone and Osteochondral Repair: Mechanisms and Outcomes

**DOI:** 10.3390/ijms262211172

**Published:** 2025-11-19

**Authors:** Priyadarshini Sakthi Mohan, Nazia Binti Abdul Majid, Raden Joko Kuncoroningrat Susilo, Wijenthiran Kunasekaran, Tan Li Jin, Lee Siew Ee, Chua Kok Seng, Gopinath Venkatraman

**Affiliations:** 1 Department of Pharmaceutical Chemistry, Faculty of Pharmacy, Universiti Malaya, Kuala Lumpur 50603, Malaysia; priyadarshini@um.edu.my; 2 Institute of Biological Sciences, Faculty of Science, Universiti Malaya, Kuala Lumpur 50603, Malaysia; nazia@um.edu.my; 3 Nanotechnology Engineering, Faculty of Advance Technology and Multidiscipline, Universitas Airlangga, Surabaya 60115, Indonesia; 4 Nano Biologics Research Centre (NBRC), WARI Technologies Sdn Bhd, 2A-2, Galleria Cyberjaya, Jalan Teknokrat 6, Cyber 5, Cyberjaya 63000, Malaysia; wijen@wari.tech (W.K.); lijin@wari.tech (T.L.J.); siewee@wari.tech (L.S.E.); petercks@gmail.com (C.K.S.); 5 Gleneagles Hospital, 282 & 286, Jalan Ampang, Kuala Lumpur 50450, Malaysia; 6 Universiti Malaya Centre for Proteomics Research (UMCPR), Universiti Malaya, Kuala Lumpur 50603, Malaysia; 7 Department of Biochemistry, Saveetha Dental College, Saveetha Institute of Medical@Technical Sciences, Saveetha University, Chennai 600077, India

**Keywords:** biocompatibility, bone repair, in vitro, exosomes, osteoarthritis, osteochondral regeneration

## Abstract

Critically sized bone defects remain a global health and economic burden, and biomaterials associated with stem cell therapy have been widely applied as a significant strategy for bone regeneration. Due to limitations related to cell survivability, immune rejection, and transplantation at the defective bone site, the improved therapeutic outcomes of stem cells are achieved through paracrine actions, which involve the secretion of extracellular vesicles (EVs) and/or other factors. Ultra-small, nano-sized exosomes (Exos) of endosomal origin have demonstrated promising potential for bone regeneration through partially revealed intercellular communication. However, the real-time feasibility before clinical trials remains unknown. The current report aims to provide an overview of the various stem cell-derived exosomes in treating bone and cartilage defects, including osteoarthritis (OA) and osteochondral defect (OCD), and optimize the yield of Exos with enhanced tissue engineering potentials. Additionally, the encapsulation of Exos with various bioactive molecules to enhance therapeutic efficacy, their functionalization with biocompatible scaffolds to promote sustained release in the defective cellular microenvironment, and the molecular functions of Exos were investigated.

## 1. Introduction

Bone, cartilage, and neuronal defects have a profound impact on physiological function, significantly decreasing the quality of life. Advancements in stem cell-based treatment modalities have been applied extensively in recent decades, offering new therapeutic options for patients with musculoskeletal and neurological disorders, as well as for neuronal repair [1,2,3]. Osteoarthritis (OA) is the most common chronic joint disease, characterized by the progressive degeneration of articular cartilage, leading to joint pain, stiffness, and reduced mobility [4]. The therapeutic potential of MSCs is driven by their ability to migrate to injury sites, differentiate into various cell types, and secrete growth factors and cytokines that promote tissue maintenance and repair [5,6]. Although therapeutic options are widely accepted, several risks have been reported, including low engraftment and viability after injection, as well as thrombus formation and pulmonary embolism resulting from cell accumulation in the lung [7]. Additionally, the accumulation of undifferentiated human pluripotent stem cells (hPMSCs) in the body leads to the risk of teratoma [8]. Long-term in vitro proliferation of MSCs can lead to genetic instability, chromosomal aberrations, and an increased risk of malignant transformation [9]. Therefore, the establishment of cell-free therapeutic molecules offers similar advancements to those of MSCs, which are paramount for safer regenerative therapies. In response to this demand, Exos derived from almost all cell types have been considered a safer and significant alternative for cell-based therapy [10,11]. MSC-derived Exos (MSC-Exos) exhibit similar functions to MSCs, such as tissue regeneration, inflammation modulation, and immune regulation [12]. Exos are small extracellular vesicles (sEVs), typically 30–150 nm in diameter, released by cells through the fusion of multivesicular bodies with the cell membrane. They have a lipid bilayer structure that protects their contents, which include regulatory proteins, signalling lipids, nucleic acids (e.g., miRNAs, mRNAs), and epigenetic modulators, which may vary based on the parental cell type, differentiation profile, and microenvironmental stimuli [13,14]. Exosomes play a critical role in intercellular communication by delivering their cargo to target cells via membrane fusion or endocytosis [15]. Exos play crucial roles in patho-physiological processes, including tissue regeneration, immune modulation, and cell proliferation and differentiation [16]. They act as “molecule carriers” and have potential applications in diagnostics, therapeutics, anti-tumour therapy, and drug delivery [17,18]. Alginate (ALG), hyaluronic acid (HA), and fibrin glue (FG) are widely used in bone tissue engineering (BTE) and serve as delivery systems for therapeutic agents, such as MSC-derived exosomes, due to their high biocompatibility, improved mechanical strength, and porosity [19,20]. In regenerative medicine, particularly in the treatment of OCD, bone defects, OA, and wound healing, Exos are involved in processes such as tissue regeneration, immune response regulation, soft tissue repair, dental pulp regeneration, and the treatment of periodontal disease [21]. Therefore, in this review, we discuss novel and effective therapeutic approaches urgently needed to promote the regeneration of bone, cartilage, and osteochondral tissue.

## 2. The Role of Different Cell-Derived Exosomes for Bone Regeneration

### 2.1. Stem Cell-Derived Exos

**Figure 1:**
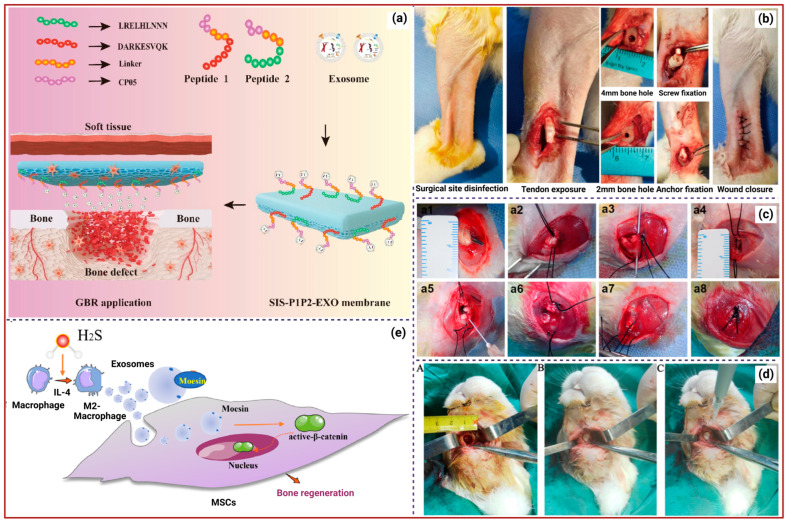
(**a**). The preparation of the SIS–P1P2-Exos membrane for effective bone repair [26]. (**b**). GelMA/BMSCs-Exo insertion with an interference screw or suture anchor for treating tendon–bone injuries in a rabbit model [29]. (**c**). The sequential surgical-insertion procedure of FG-PRP-Exos. (**a1**) Blunt dissection of the infraspinatus tendon was performed, (**a2**) followed by threading of 3-0 Ethibond sutures through the tendon and (**a3**) creation of the insertion site and syringe needle (5mL) to create double rows of bony tunnels at the higher tuberosity of the humerus. (**a4**) Passed two sutures through the bone tunnel from inside to outside. (**a5**) Administered various treatments per procedure using a double syringe. (**a6**) Allowed FG-PRP-Exos to get to the bone–tendon interface. (**a7**) Completely covered the infraspinatus tendon with the gel formulation and (**a8**) conducted gross observation after the modified Mason–Allen suture [31]. (**d**). Induced critical mandibular bone defect in rabbits (**A**), inserted the nHA/CS/PLGA-Exos scaffolds into the defect site (**B**), and loaded BMSCs into the defect cavity (**C**) [53]. (**e**). The role of H2S-modified M2-Exos in osteogenic differentiation of MSCs. H2S promotes the polarization of macrophages into the M2 subtype and enhances the level of exosomal protein moesin in M2 macrophages, thereby facilitating the uptake of Exos by MSCs. The internalized Exos activate the β-catenin signalling pathway in MSCs and enhance osteogenic differentiation, leading to improved bone regeneration [68].

A recent study revealed that bone marrow mesenchymal stem cell-derived exosomes (BMSC-Exos) loaded into HA-based 3D-printed hydrogel (HG) scaffolds exhibited superior bone regeneration potential. In vivo experiments using male adult Sprague-Dawley (SD) rats showed that the Exos and oxygen released from the scaffold regulate the inflammatory microenvironment by reducing pro-inflammatory factors (e.g., TNF-α, IL-1β, IL-6), promoting macrophage polarization to the M2 phenotype, while calcium ions released from the scaffold enhance the expression of osteogenic genes such as alkaline phosphatase (ALP), Runx2, and osteocalcin (OCN), promoting the differentiation of BMSCs into osteoblasts (OBs). This reduces chronic inflammation and improves oxygen delivery, enhancing cellular metabolism and accelerating bone tissue repair [22]. A recent study concluded that the 3D-cultured model of human periodontal ligament stem cell (hPDLSC)-derived Exos (hPDLSCs-Exos) enhanced bone repair potential by activating YAP signalling pathways, which boosts osteogenesis, cell proliferation, migration, and survival, ultimately leading to effective bone regeneration compared to traditional 2D culture-derived Exos [23]. For instance, Xiang et al. developed the 3D-BMSC-Exo/HG scaffold, in which Exos enhance bone regeneration by addressing hypoxia and oxidative stress in bone defects, while HG improves the bone microenvironment and promotes osteogenesis. Oxidative stress is the primary impediment to bone repair, underscoring the need for effective ROS scavenging strategies. Hence, 3D-Exos were modified with superparamagnetic Iron oxide nanoparticles (SPIONs) (3D-SExos) for enhanced reactive oxygen species (ROS) scavenging, followed by the incorporation of bone-targeting peptide-modified GelMA (3D-SExos/DGel). 3D-SExos/DGel reduced ROS levels in BMSC by 49% compared to the control and enhanced OB differentiation in vitro [24]. Adipose-derived stem cell (ADSC)-derived Exos (ADSC-Exos) cultured in a 3D environment (3D-Exos) demonstrated superior effects in enhancing bone mineral density (BMD), bone volume (BV) fraction, and other bone-related parameters compared to 2D-Exos. 3D-Exos facilitated OB differentiation and maturation, leading to increased ALP activity, calcium nodule formation, and upregulation of osteogenesis-related genes. It has also been identified that 3D-Exos has a distinct miRNA profile, with upregulated miRNAs promoting osteogenesis (miR-3648) and downregulated miRNAs inhibiting osteogenesis (let-7i-5p) related to bone development, ossification, and OB differentiation [25]. For instance, collagen-binding peptides (targeting collagen I (COL-I) or collagen III (COL-III)) linked with the exosomal capture peptide CP05, bound to the small intestinal submucosa (SIS), were found to recruit Exos effectively. The engineered recombinant peptides (SIS–P1P2-Exos) significantly enhanced the osteogenic differentiation of BMSCs, evidenced by increased expression of OCN, ALP, bone morphogenetic protein-2 (BMP2), and osteopontin (OPN), and facilitated the activation of the PI3K/Akt signalling pathway. In vivo studies showed that the SIS–P1P2-Exos exhibited superior bone regeneration capabilities, effectively reconstructing bone defects within 12 weeks (Figure 1b) [26]. ALG-HA/MSC-Exos HG showed higher levels of ALP expression, indicating enhanced osteogenic differentiation, BMSC engraftment, and mineralization. Additionally, the HG facilitated tube formation by human umbilical vein endothelial cells (hUVECs), with increased branching points and capillary length, and showed notably higher migration rates of hUVECs and BMSCs. The in vivo results of ALG-HA/MSC-Exos showed the highest bone-tissue-volume-to-total-tissue-volume (BV/TV) ratio and BMD, and highly expressed RUNX-2 and OCN, indicating significant bone defect repair [27].

For instance, Hypoxic umbilical cord stem cell-derived Exos (Hy-UCMSC-Exos) have high therapeutic potential and are enriched with miR-126. These Exos are more readily taken up by endothelial cells compared to those derived under normoxic conditions. Likewise, exosomal miR-126 is transferred to hUVECs, where it suppresses SPRED1 expression, activating the Ras/Erk signalling pathway and promoting endothelial cell proliferation, angiogenesis, and migration. This enhanced angiogenesis contributes to improved vascularization at the fracture site, facilitating nutrient delivery and bone regeneration [28]. Furthermore, GelMA-encapsulated BMSCs-Exos decreased the expression of IL-1β, IL-6, and TNF-α, and increased anti-inflammatory factors IL-10 and transforming growth factor-β (TGF-β), facilitating the formation of new chondrocytes and cartilage-like structures at the tendon–bone interface (TBI) (Figure 1e). Although no significant differences in the inflammatory response were observed between the two fixation methods, the interference screw provided stronger fixation, while the suture anchor showed greater tissue regeneration [29]. The therapeutic efficacy of BMSC-Exos was enhanced by reversibly binding them with tannic acid-modified sulfonated polyetheretherketone (TA-SPEEK) implants. The implant modulates the host immune response by promoting macrophage M2 polarization via the NF-κB pathway to create a favourable environment for bone regeneration. The sustained release of Exos fosters the expression of osteogenesis-related genes and proteins, including COL-I, Runx2, OPN, and OCN. The Exos-loaded TA-SPEEK implantation in rat femoral drilling models demonstrated new bone formation around the implant site and minimized fibrous tissue formation, ensuring better integration with the bone [30]. Platelet-rich plasma-derived Exos (PRP-Exos) induce changes in tendon stem/progenitor cells (TSPCs), promoting their proliferation, migration, and differentiation into tendon and cartilage lineages, as well as angiogenesis. The formation of new blood vessels, which improves vascular function and nutrient delivery to the injured area, is crucial for the healing of rotator cuff tears (RCTs). Furthermore, PRP-Exos activate the Akt/Bad/Bcl-2 pathways, regulate inflammatory responses, and deliver platelet-derived growth factor (PDGF), vascular endothelial growth factor (VEGF), and fibroblast growth factor (FGF) to promote tissue repair, including fibrocartilage regeneration and cellular adhesion [31]. Exos derived from a specific subpopulation of adipose-derived chondrogenic stem/progenitor cells (Sub-Exos) enhance chondrogenic differentiation of hBMSCs, extracellular matrix (ECM) synthesis, and anti-inflammatory responses, thereby driving tissue repair at the TBI. In vivo studies demonstrated improved collagen organization and subchondral bone formation at the TBI, indicating superior healing in chronic RCTs [32]. Another study revealed that PRP-exos significantly enhance the proliferation and differentiation of tendon-derived stem cells (TDSCs), leading to increased production of tendon-specific markers, including COL-II, SOX-9, and TIMP-1. In vivo experiments using rabbit models demonstrated improved tissue arrangement, reduced inflammation, and accelerated healing at the TBI following PRP-Exos treatment [33]. For instance, dermal fibroblast-derived Exos (DF-Exos) combined with FG enhanced early mRNA expression of COL-1A1, COL-3A1, and ACAN, which are critical for ECM and fibrocartilage formation. The in vivo study demonstrated improved collagen fibre continuity, density, and orientation, as well as the formation of a fibrocartilage layer, leading to a more mature bone–tendon interface structure in a rabbit model of chronic RCT [34].

Exos derived from stem cells of human exfoliated deciduous teeth (SHED-Exos) enhance osteogenic differentiation and regulate the apoptosis mechanism of human periodontal ligament cells (hPDLCs) to facilitate tissue regeneration. The specific miRNAs of SHED-Exos influence signalling pathways such as MAPK and Wnt, which are involved in cell survival, proliferation, apoptosis, and differentiation. Moreover, they modulate the mTOR, AMPK, FoxO, and PI3K-Akt pathways, which are associated with cellular processes such as energy expenditure, oxidative stress, and osteogenesis [35]. Three-dimensionally printed porous Ti6Al4V scaffolds decorated with Schwann cell-derived Exos (SC-Exo) demonstrated that Exos enhance BMSC migration, proliferation, and differentiation, improving titanium alloy scaffold efficacy in bone repair. The report suggested that the SC-Exos effectively promote bone regeneration and enhance the effectiveness of porous Ti6Al4V scaffolds, offering a new therapeutic strategy for bone defects [36]. Sun et al. demonstrated that BMP2-overexpressed Exos (BMP2-Exos) are encapsulated within a GelMA-HG, providing a porous structure that mimics the ECM and facilitating cell communication, nutrient transport, and sustained delivery of Exos at the bone defect site. BMP2-Exos significantly enhance the proliferation and increase the expression of ALP, COL-I, BMP2, and RUNX2. Furthermore, BMP2-Exos-GelMA-HG promotes the formation of new bone tissue in a mouse model of a cranial defect [37]. A recent study utilized small extracellular vesicles (sEVs), a subset of EVs often referred to as Exos, loaded with the therapeutic genetic materials, including human VEGF-A and BMP-2 mRNAs, using a track-etched membrane-based nanoelectroporation (TM-nanoEP) system. The HG-encapsulated sEVs (HG-sEVs) were injected into rats with critical-size femoral defects, demonstrating controlled, localized release of therapeutic mRNAs for VEGF-A and BMP-2, which synergistically promote angiogenesis and osteogenesis, effectively addressing critical-size bone defects [38]. For instance, the hUCMSC-sEVs encapsulated in bio-glass (BG) scaffolds with HG coatings (BG-HG-sEVs) exhibited a slow, sustained release of sEVs, which are taken up by BMSCs and hUVECs. The delivery of miR-23a-3p via sEVs to recipient cells targets the 3′UTR of PTEN, suppressing its expression and activating the PTEN/AKT signalling pathway to promote bone and blood vessel formation. Furthermore, the in vivo study demonstrated that the BG-HG-sEV scaffold effectively promotes bone regeneration in critical-size calvarial bone defects in mice. The Micro-CT and histological evaluations showed increased BV/TV, BMD, and vascularization in the defect area, indicating high-quality bone repair [39]. Wu et al. demonstrated a thermosensitive HG encapsulated with BMSC-sEVs (tHG-BMSC-sEVs), which is injectable and forms a gel at body temperature. The BMSC-sEVs contain exosomal miR-21 targeting SPRY2, a gene that negatively regulates angiogenesis, thereby enhancing the expression of angiogenic factors such as VEGF, bFGF, and ANG-1. The released sEVs are internalized by hUVECs and BMSCs, promoting osteogenic differentiation, cell proliferation, migration, and tube formation. Furthermore, they enhance angiogenesis, which is critical for bone regeneration. In a rat model of calvarial defects, the tHG-BMSC-sEVs demonstrated improved bone healing and new vessel formation [40]. Given the current therapeutic challenges and limitations, these advancements could establish miRNA-based sEV therapies as viable and effective treatments for bone-related diseases and injuries [41]. Furthermore, electrical stimulation is recognized as a promising method to enhance osteogenesis and accelerate bone regeneration. However, its clinical potential is hindered by the complexity of procedures and the need for invasive equipment [42]. For instance, a study investigated the therapeutic efficacy of Exos derived from electrically stimulated BMSCs (Ele-Exos) in chondroitin sulfate methacrylate (CSMA) hydrogels (CSMA + Ele-exo), where the electrical stimulation significantly induced osteogenesis, mineralization, and enrichment of oxidative phosphorylation, thus promoting bone regeneration. Together, the findings suggest that electrical stimulation enhances the therapeutic potential of Exos for bone regeneration, opening a new platform to address bone defects [43]. Although Exos from MSCs have numerous therapeutic properties, their production yield is low. Traditional electrical stimulation methods are costly and often damage cells due to electrolysis. To overcome these challenges, a novel self-powered electrical system (SES) using triboelectric nanogenerators (TENGs) was developed, increasing Exos production by up to 3.2 times without affecting bioactivity [44]. In another report, BMSCs treated with mesoporous bioactive glass (MBG) were found to enhance the production of Exos (MBG-Exos) and their biological functions. MBG-Exos promoted the proliferation, osteogenic differentiation, and mineralization of OBs in vitro. In vivo, results showed that MBG-Exos promoted vascularization at the defect site and induced M2 macrophage polarization (anti-inflammatory) while inhibiting M1 macrophage polarization (pro-inflammatory), creating a favourable immune microenvironment in cranial defects in rats [45].

MSC-Exos demonstrated enhanced bone tissue regenerative potential by facilitating angiogenesis and osteogenesis via the delivery of proteins, lipids, and epigenetic modulators [46]. However, insufficient therapeutic efficacy and unstable immunoregulatory capacity are key challenges that need to be addressed. Hence, a recent attempt, shown in MSC-Exos, was encapsulated with curcumin (Cur) in a bisphosphonate-modified HG (CE@BP-HG) microsphere system to investigate its therapeutic efficacy. Cur, a natural compound derived from turmeric, exhibits anti-inflammatory, antioxidant, and osteogenic properties that effectively suppress TNF-α and IL-6 [47], which are crucial in delayed bone repair. Moreover, Cur’s bioavailability is enhanced when it is encapsulated in Exos. The study results indicate that the CE@BP-Gel microspheres can be effectively applied in cartilage repair and chronic wound healing [48]. Wang et al. revealed that the hMSCs-Exos released in the late stage of osteogenic differentiation contain high levels of osteogenesis-related microRNAs, such as miR-21, which promote osteogenic differentiation, and show reduced levels of negative regulators like miR-31, miR-221, and miR-144 and ECM mineralization compared to the expansion stage and early differentiation, in which poor osteogenic differentiation was recorded [49]. Additionally, the limited osteogenic potential of MSC-Exos was enhanced by developing Exos-mimetics (Ems) by integrating osteogenically induced MSC spheroids into self-healing, injectable HGs, thereby improving bone regeneration. The in vivo assessment revealed that the Ems-HG system alters miRNA expression related to the Wnt/β-catenin and Notch signalling pathways, thereby enhancing calvarial bone tissue regeneration in the mouse model [50]. Wang et al. developed an exosome-capturing scaffold (ECS) model that attracts and anchors neutrophil-derived Exos (PMN-Exos) through electrostatic and lipophilic interactions. The PMN-Exos enriched ECS stimulated endothelial progenitor cell (EPC) proliferation, a critical factor in vasculogenesis and angiogenesis, through the miR455-3p/Smad4 pathway. In vivo studies demonstrated that ECS significantly improved bone regeneration in rat and mini-pig mandibular defect models [51].

A recent study reported that ADSC-Exos enhanced the proliferation, migration, and osteogenic differentiation of BMSCs, as confirmed by the expression of related genes, including RUNX2, OSX, OCN, OPN, and COL-I. Furthermore, ADSC-Exos loaded into a gelatin sponge coated with polydopamine (GS-PDA) ensured their prolonged release. The in vivo study in a rat femur defect model showed that the GS-PDA-Exos scaffold significantly improved BMD, BV/TV, trabecular thickness (Tb.Th), and trabecular separation (Tb.Sp) [52]. Similarly, the controlled release of ADSC-Exos in the nHA/CS/PLGA scaffolds increased mineralized nodule formation and ALP activity, and elevated mRNA expression of osteogenic markers like COL-1A1 and RUNX2. In vivo studies have demonstrated that scaffolds loaded with ADSC-Exos and BMSCs effectively repair maxillofacial bone defects in rabbits (Figure 1d), resulting in the formation of new bone and blood vessels [53]. Another report revealed that the BMSC-Exos improve angiogenesis, immunoregulation, and intercellular communication. Furthermore, they enhance myogenic differentiation of myocytes and stimulate macrophage polarization toward the regenerative M2 phenotype while reducing pro-inflammatory M1 macrophages, thereby collectively guiding bone–muscle regeneration [54]. For instance, rat BMSC-derived osteoinductive Exos (rBMSC-OI-Exos) contain multi-component miRNAs that target Acvr2b/Acvr1 receptors and regulate the competitive balance between Bmpr2 and Acvr2b receptors, favouring Bmpr2-mediated Smad1/5/9 phosphorylation, which activates the canonical BMP/Smad signalling pathway. Moreover, the in vivo rat cranial defect model treated with the rBMSC-OI-Exos immobilized MBG (rBMSC-OI-Exos-MBG) scaffold demonstrated enhanced bone formation, bone regeneration, and upregulated BMP-2 and pSmad1/5/9 expression, confirming the activation of the BMP/Smad pathway [55]. BMSC-Exos were loaded into a porous HG, which promoted osteogenic gene expression, including COL-I, OCN, and RUNX2, and stimulated the expression of angiogenesis-related proteins, such as CD31 and VEGF, in hUVECs. In a rat model of cranial defects, BMSCs-Exos-HG significantly promoted bone regeneration, showing an increased BV/TV ratio [56].

### 2.2. Macrophage-Derived Exos (M-Exos)

Macrophages are versatile immune cells that play a central role in the immune system. They exhibit functional plasticity and can polarize into different phenotypes, such as pro-inflammatory M1 macrophages and anti-inflammatory M2 macrophages, based on microenvironmental signals [57]. M-Exos, particularly those from M2 macrophages, play a significant role in bone repair by promoting osteogenesis, angiogenesis, and immune modulation due to their unique cargoes [58,59]. Their therapeutic potential stems from their ability to deliver miRNAs that regulate cellular processes crucial to bone repair. For instance, M2–exosomal miRNA-26a-5p enhances the expression of ALP, RUNX-2, OPN, and COL-II, which are essential for bone formation and repair processes. Additionally, miRNA-26a-5p inhibits adipogenic differentiation, further supporting its role in bone regeneration [60]. In another study, M2-Exos were engineered to express miR-365-2-5p (M2-Exos/miR-365-2-5p) to address challenges associated with poor targeting and insufficient therapeutic concentrations in exosomal therapy. M2-Exos/miR-365-2-5p were effectively internalized by OBs, which significantly enhanced osteogenesis by targeting the OLFML1 gene [61]. The OLFML1 gene encodes the protein Olfactomedin-like 1, a negative regulator of OB differentiation which inhibits the Hippo signalling pathway by suppressing the nuclear translocation of Yes-associated protein (YAP). Knockdown of OLFML1 leads to increased YAP accumulation in the nucleus, promoting OB differentiation and mineralization. Conversely, overexpression of OLFML1 inhibits OB differentiation and mineralization [62]. A recent report demonstrated that under shear stress, M2-Exos downregulate miR-423-5p, a negative regulator of osteogenesis that binds specifically to BMP-2, thereby suppressing BMP-2 expression. Downregulation of miR-423-5p in Exos relieves this suppression, leading to increased BMP-2 levels and thereby promoting MMSC osteogenesis by activating MAPK and RAS signalling pathways. This leads to upregulation of osteogenic markers, including RUNX2, ALP, and BMP-2 [63]. A novel biomimetic periosteum, developed using M2-Exos functionalized with BMSC-specific aptamers (PEC-Apt-NP-Exos) via coaxial electrospinning, effectively promotes BMSC migration and osteogenic differentiation through the Rap1/PI3K/AKT signalling pathway. In vivo studies reveal that M2 macrophages were more abundant in PEC-Apt-NP-Exos (76.60 ± 5.03) than in PEC (50.60 ± 6.47). The M2/M1 ratio in PEC-Apt-NP-Exos was 1.80 ± 0.12, indicating effective polarization and endogenous BMSC recruitment, thereby promoting new bone formation in large-bone-defect repairs [64].

Furthermore, BMP2/M-Exos integrated with a titanium nanotube (Ti-Nt) scaffold effectively stimulate the expression of osteogenesis-related markers (ALP, BMP2, and Runx2), promoting early OB differentiation. The scaffold activates autophagy in hBMSCs, maintains cellular homeostasis, and increases levels of pro-osteogenic cytokines (IL-17A, IL-19, RANTES) that support bone regeneration [65]. β-TCP bioceramic-stimulated M-Exos contain specific miRNA cargos such as pro-osteogenic mmu-miR-330-3p and mmu-miR-338-3p, pro-angiogenic mmu-miR-18a-5p and mmu-miR-25a-3p, and anti-inflammatory mmu-miR-125a-5p and mmu-miR-130b-5p. The prolonged release of M-Exos from the 3D-printed porous scaffolds regulates MAPK, NFκB, and BMP2/Smad signalling pathways, which are critical for tissue regeneration [66]. M2-Exos promote osteogenic differentiation of MSCs by activating the Wnt/β-catenin signalling pathway, thereby enhancing β-catenin nuclear translocation. This process regulates the expression of Runx2, COL-I, and OPN, promoting bone formation [67]. Furthermore, H2S-pretreated M2-Exos facilitate the polarization of macrophages into the M2 subtype, which is anti-inflammatory and supports tissue repair. H2S alters the protein profile of M2-Exos, significantly enriching moesin, which facilitates Exos’ endocytosis into MSCs and enhances their uptake. Once internalized, the Exos activate the β-catenin signalling pathway in MSCs, which is crucial for osteogenic differentiation and bone regeneration, as represented in Figure 1a [68]. Additionally, a lipid-metabolizing enzyme, sphingomyelin phosphodiesterase 3 (Smpd3) in bone, plays a vital role in the bone regeneration process [69]. In certain metabolic conditions, the bone regenerative potential is significantly low, especially in Type 2 diabetes mellitus (T2DM) [70]. In such conditions, Exos-Smpd3@Ns enhance the osteogenic differentiation of BMSCs by promoting autophagy and increasing osteogenic marker expression while also shifting macrophage polarization toward an anti-inflammatory profile. Additionally, they reduce reactive oxygen species (ROS) levels and alleviate oxidative stress by maintaining mitochondrial membrane potential, creating a conducive environment for bone healing [71].

### 2.3. OB-Exos and Osteoclast-Derived Exos (OC-Exos)

OBs play a crucial role in bone formation, and Wnt signalling is essential for OB differentiation and bone development [72]. A recent study demonstrated that OB-Exos promote MSCs’ differentiation into OBs by activating key signalling pathways, including Wnt/β-catenin and BMP/Smad. The exosomal microRNAs, such as miR-21 and miR-214, enhance the expression of ALPL, RUNX2, and OPN, leading to mineralization and bone matrix formation. Furthermore, they promote vascularization in the bone microenvironment through VEGF/ERK1/2 pathways and release the angiogenic factor matrix metalloproteinase-2 (MMP2). Furthermore, it influences macrophage polarization and reduces inflammation, which is critical for bone healing [73]. For instance, the MMP2 present in MOB-Exos activates the VEGF/Erk1/2 signalling pathway in ECs, leading to increased expression of VEGF, phosphorylated VEGF receptor 2 (pVEGFR2), and phosphorylated extracellular regulated protein kinases 1/2 (pErk1/2). These activations contribute to the enhanced angiogenesis observed in ECs [74]. OB-Exos regulate OC differentiation by transferring miR-503-3p to OC progenitor cells, thereby inhibiting osteoclastogenesis by downregulating Hpse (heparanase) expression. This mechanism highlights the role of OB-Exos in bone remodelling and cellular communication [75]. Chen et al. demonstrated that Auto-OB-Exos treatment in ovariectomized (OVX) mice increased bone mineral density (BMD), improved bone microstructure, reduced OC activity, and upregulated RUNX2, OSX, Plzf, Dlx5, and ALP. It altered the gut microbiota by increasing the abundance of Lactobacillus, which regulates bone metabolism, and decreasing harmful bacteria such as Dubosiella and Faecalibaculum. Furthermore, the Exos suppressed bilirubin production, thereby promoting OB differentiation and bone formation, and alleviating OP progression [76]. OB-Exos inhibit osteosarcoma (OS) cell proliferation, promote mineralization, and upregulate osteogenic markers like osteonectin (ON), bone sialoprotein (BSP), RUNX2, and OPN while downregulating URG4 expression. Furthermore, OB-Exos upregulate Wnt inhibitory factor 1 (WIF1), which inhibits the Wnt/β-catenin signalling pathway, leading to decreased nuclear accumulation of β-catenin and increased β-catenin phosphorylation, thereby suppressing the pathway’s activity. Cyclin D1, a downstream target of the Wnt/β-catenin pathway involved in cell proliferation, is also downregulated. These specific roles of OB-Exos make them a potential therapeutic tool for treating OS [77]. Similarly, Kim et al. demonstrated that the transfection of matured OB-derived EVs enriched with miR-125b, a tumour-suppressor miRNA, into MM cells suppresses the expression of interferon regulatory factor 4 (IRF4) and MYC, leading to MM cell apoptosis. This effect is specific to MM cells, as EVs do not affect the viability of normal cells, such as peripheral blood mononuclear cells, bone marrow stromal cells, or OCs. This process establishes a non-permissive niche for MM cells in the bone marrow [78]. A recent study demonstrated that the Exos derived from genetically modified WIF1-overexpressing MC3T3-E1 OB cells (WIF1oe-OB-Exos) promote bone formation during the late stage of OB differentiation by suppressing β-catenin levels, which helps regulate mineralization and OB maturation. Inhibiting Wnt signalling has been shown to activate mitophagy, a process that maintains mitochondrial health and promotes the secretion of EVs that contain osteogenic factors, thereby enhancing bone formation. In vivo results showed that the WIF1oe-OB-Exos injection in OVX mouse models significantly ameliorated bone loss. The Micro-CT analysis showed improved bone mass retention, including increased BV/TV, Tb.N, and Tb.Th, along with reduced Tb.Sp. Targeting Wnt signalling at specific stages may offer therapeutic potential for conditions such as OP, but further research is needed to understand the mechanisms and optimize treatment strategies [79]. In contrast, the long non-coding RNA (LncRNA) AW011738 in OC-Exos inhibits OB differentiation in MC3T3-E1 cells and reduces bone formation. The mechanism involves the LncRNA AW011738/miR-24-2-5p/TREM1 axis, in which AW011738 suppresses miR-24-2-5p, thereby increasing TREM1 expression. TREM1 further inhibits OB differentiation and promotes bone loss. The report also showed that OC-Exos decrease osteogenesis-related markers, weaken osteogenic differentiation, and exacerbate OP in OVX mice [80]. A recent study demonstrated that under compression stress, the miRNA profiles of OC-EXOs were altered, with miR-223-5p and miR-181a-5p downregulated and miR-133a-3p, miR-203a-3p, miR-106a-5p, and miR-331-3p upregulated. These Exos inhibited OB differentiation in human periodontal ligament stem cells (hPDLSCs). Moreover, the altered miRNA profiles were linked to signalling pathways, including MAPK, mTOR, and insulin signalling, which are known to regulate bone formation and remodelling [81].

## 3. Exosomes for Osteoarthritis Treatment

**Figure 2:**
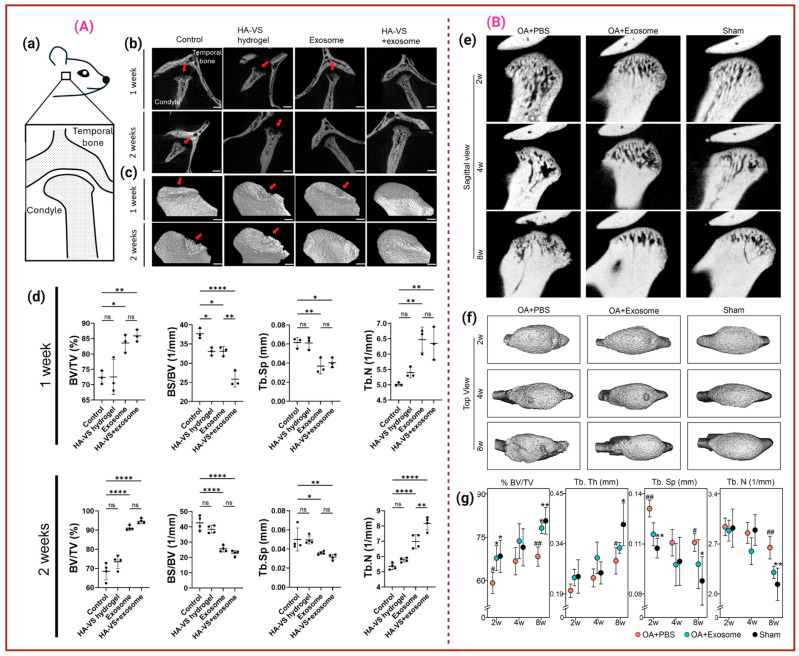
(**A**). Representing the Sagittal view of the rat TMJ model (**a**). Sagittal and 3D view of TMJ treated with the control, HA-VS/HG, hDPSC-Exos, and HA-VS/Exos after 1 and 2 weeks, in which red arrows indicate the bone deterioration (**b**,**c**). MicroCT determination of BV/TV, bone surface (BS) to volume (BS/BV, mm^−1^) Tb.Sp, and Tb number (Tb.N, mm^−1^); * *p* < 0.05, ** *p* < 0.01, **** *p* < 0.0001, and ‘ns’ stands for not significant (**d**) [87]. (**B**). Micro-CT analysis of rat TMJs treated with PBS, Exos, and sham at 2, 4, and 8 weeks. Sagittal and top view of the condyles (**e**,**f**). Representative images (*n* = 6–8). (**g**) Ratios of BV/TV, Tb·Th, Tb.Sp, and Tb·N. * *p* <0.05 and ** *p* < 0.01 compared to OA + PBS gp; # *p* < 0.05 and ## *p* < 0.01 compared to sham gp. *n* = 6–8/gp [88].

**Figure 3:**
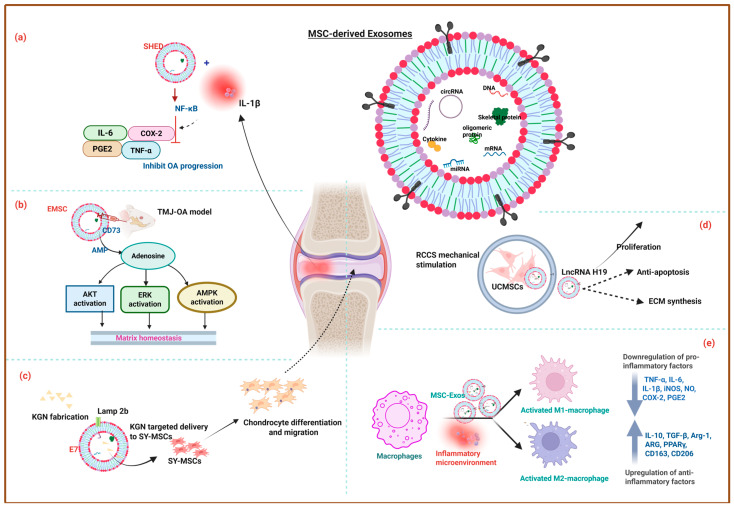
Schematic diagram of MSC-Exos and their tissue engineering potential in treating osteoarthritis through (**a**) Matrix homeostasis [88], (**b**). activation of anti-inflammatory signalling pathway [89], (**c**). improving chondrocytes proliferation, migration [93], and treating osteochondral defect through (**d**). extracellular matrix synthesis [94], and (**e**). macrophage polarization [95]. The figure was created using the Biorender Academic Individual License.

The typical OA treatment strategy involves joint mobilization, exercise therapy, Transcutaneous electrical nerve stimulation, ultrasound therapy, Infrared therapy, and lifestyle modifications to improve blood circulation, reduce pain, and improve joint function [82,83]. However, recent investigations reveal that Exos derived from polydactyly-BMSCs (pBMSC-Exos) stimulate chondrocyte proliferation via BMP4 signalling. The in vivo results showed that pBMSC-Exos significantly reduced cartilage damage and improved joint health in a collagenase-induced OA mouse model, making them a vital component of OA treatment [84]. For instance, UCMSC-Exos firmly preserve the ECM of hyaline cartilage, while significantly downregulating inflammatory markers (TNF-α, iNOS, NLRP3) and increasing the expression of the anti-inflammatory marker Arg-1 in macrophages and synovial fibroblasts. Together, the in vivo rat OA model treatment demonstrated that UCMSC-Exos almost eliminate degenerative processes in joint tissues [85]. Notably, UCMSCs cultured in Exos-depleted FBS exhibited better chondrogenic potential in both in vitro and in vivo settings compared to FBS-cultured UCMSCs. This may be due to the removal of Exos from FBS, which retains MSC characteristics and provides a more controlled environment for chondrogenic differentiation [86]. Dental pulp stem cell-derived Exos (DPSC-Exos) promote anti-inflammatory, tissue repair properties, attenuate subchondral bone deterioration, effectively moderate temporomandibular joint osteoarthritis (TMJ-OA) progression, and protect joint integrity in a rat TMJ-OA model. Moreover, the protective effects were enhanced when Exos were delivered within the HG system of vinyl sulfone-modified HA (HA-VS/HG) due to the sustained Exos release. Significant subchondral bone deterioration in the control and HA-VS/HG group (gp), while TMJs treated with hDPSC-Exos alone and HA-VS/HG-Exos demonstrated better maintenance of joint structure, including smoother and more continuous condylar surfaces, as shown in Figure 2A [87]. Zhang et al. demonstrated that the Immortalized human embryonic stem cell-derived E1-MYC 16.3 Exos (EMSC-Exos) suppress the expression of pain-associated genes and pro-inflammatory markers, such as IL-1β, and enhance matrix synthesis by suppressing enzymes like MMP13 and ADAMTS5, thereby reducing matrix degradation. The in vivo micro-CT analysis demonstrated that EMSC-Exos treatment at 2 weeks showed early attenuation of bone loss, with an increased BV/TV compared to the PBS-treated gp, followed by increased BV/TV and Tb·Th with decreased Tb·Sp and Tb·N in the EMSC-Exos gp after 8 weeks, as highlighted in Figure 2B. Additionally, EMSC-Exos activate AKT, ERK, and AMPK signalling pathways to regulate matrix homeostasis, suppress inflammation, and enhance tissue repair, as shown in Figure 3a. These mechanisms collectively restore joint homeostasis, alleviate TMJ degeneration, and promote repair and regeneration of TMJ tissues [88]. Lin et al. found that the Exos from human exfoliated deciduous tooth stem cells (SHED-Exos) inhibit TNF-α, IL-6, iNOS, NO, COX-2, and PGE2, which are associated with OA progression and regulate the NF-κB pathway, a key driver of inflammation in OA. Furthermore, they promote the synthesis of COL-II and Aggrecan (ACAN) to restore cartilage integrity, while protecting chondrocytes from apoptosis and promoting their survival and proliferation, thereby aiding in cartilage repair, as represented in Figure 3b [89].

DPSC-Exos alleviate abnormal subchondral bone remodelling and reduce cortical bone sclerosis and cartilage degradation by enhancing COL-II expression and improving cartilage matrix metabolism. Furthermore, DPSC-Exos suppress the overexpression of inflammatory cytokines in synovial tissues in vivo. They inhibit OC activation by suppressing transient receptor potential vanilloid 4 (TRPV4)-mediated calcium influx, a crucial step for OC differentiation [90]. Contrastingly, OA subchondral bone-derived Exos (OA-SB-Exos) alter the phenotype of articular cartilage chondrocytes through multiple pathways, such as suppressing glycosaminoglycan (GAG) production, reducing chondrocyte-specific markers, and upregulating hypertrophic and degradative markers like COL-10, MMP13, and ADAMTS5, which affect cartilage integrity and alter the bioenergetic state by suppressing oxygen levels through miR-210-5p. Targeting miR-210-5p could offer potential therapeutic strategies for OA treatment [91]. Similarly, Wu et al. found that the synovial Exos (Sy-Exos) isolated from RA, OA, and ankylosing spondylitis (AS) co-cultured with macrophages expressed high levels of RANKL in RA-Exos, which enhanced OC differentiation even in the absence of soluble RANKL, highlighting its critical role in bone destruction associated with inflammatory arthritis (IA). This could indicate the role of Exos as potential biomarkers for evaluating joint inflammation and their role in the pathogenesis of RA. However, extensive research is recommended to explore the molecular mechanisms and therapeutic implications of exosomal contents in IA [92].

For instance, the Sy-Exos membrane protein Lamp2b, engineered to bind an MSC-binding peptide E7-encapsulated kartogenin (KGN), is specifically targeted for KGN delivery to Sy-MSCs, thereby alleviating the challenges posed by KGN’s low water solubility and its tendency to aggregate within cells. In a rat OA model, co-administration of SF-MSCs with E7-Exo/KGN demonstrated superior cartilage regeneration, as represented in Figure 3c [93]. Another study showed that hUCMSC-Exos are loaded with kartogenin KGN and TGF-β1, and coated with succinylated chitosan (sCH) to create positively charged Exos (CEKT), which are further crosslinked with oxidized chondroitin sulfate (oCS) and Wharton’s jelly (WJ) to form an HG (HG-CEKT) that mimics the ECM of cartilage. The HG recruits BMSCs and promotes their differentiation into chondrocytes. In vivo studies demonstrate that Gel-CEKT promotes extensive new cartilage formation, a smoother cartilage surface, significant chondrocyte production, and seamless integration with the subchondral bone [96]. Hypoxia-cultured ADSC-Exos (Hy-ADSC-Exos) play a significant role in alleviating OA progression in ACLT-induced OA rats. They improved knee weight-bearing function, preserved cartilage integrity, and increased normal matrix levels of GAG and COL-II. They also suppressed matrix degradation enzymes, inflammatory mediators, and markers of degenerative cartilage (COL-I) [97]. Meanwhile, BMSC-Exos and UCMSC-Exos consistently outperformed ADSC-Exos in mitigating inflammation, promoting cartilage regeneration, and inhibiting chondrocyte apoptosis in vitro. Among the three, BMSC-Exos exhibited the most pronounced therapeutic benefits, making them the most promising candidate for OA treatment [98]. Although BMSC-Exos and ADSC-Exos demonstrated promising cell-free therapeutic options for ameliorating OA, BMSCs showed superior therapeutic effects compared to those from ADSCs, making them a better choice for improving OA and serving as a potential disease-modifying treatment [99]. However, Tropoelastin (TE) pretreatment with ADSC-Exos (TE-ADSC-Exos) significantly enhances cartilage matrix synthesis and alleviates OA due to the upregulation of miR-451-5p induced by TE pretreatment. Furthermore, TE-ADSC-Exos demonstrate better outcomes in reducing cartilage damage and synovial inflammation in OA rat models compared to ADSC-Exos [100]. For instance, a recent study showed that the circRNAs in BMSC-Exos of postmenopausal osteoporosis (PMOP) patients influence the disease progression. The results revealed that five circRNAs, including hsa_circ_0009127, hsa_circ_0090759, hsa_circ_0058392, hsa_circ_0090247, and hsa_circ_0049484, were identified as potential predictive biomarkers and therapeutic targets for PMOP. Moreover, these circRNAs are implicated in pathways such as autophagy, PI3K-Akt signalling, FoxO signalling, and MAPK signalling, which are associated with PMOP pathogenesis [101]. Furthermore, the Exos release kinetics and mechanical matching for musculoskeletal repair are highlighted in Table 1.

## 4. The Role of Exos in Osteochondral Regeneration

Exos from UCMSCs cultured on 3D scaffolds (3D-UCMSC-Exos) promote cell-to-cell communication, thereby significantly enhancing tissue repair, compared to those from 2D cultures (2D-UCMSC-Exos). In vitro studies showed that 3D-UCMSC-Exos enhanced BMSC proliferation, migration, and chondrogenic differentiation compared to the 2D-Exos. In vivo experiments demonstrate that 3D-UCMSCs-Exos, when combined with the scaffolds, significantly enhance cartilage repair. This may be due to the differences in miRNA profiles between 2D-Exos and 3D-Exos, which influence cartilage repair [104]. Similarly, 3D cultures of UCMSC-Exos using a hollow-fibre bioreactor (FiberCell Systems (C2025, Frederick, MD, USA) activate the TGF-β1-dependent Smad2/3 signalling pathway, thereby promoting chondrocyte proliferation, differentiation, and matrix synthesis while inhibiting hypertrophy and ossification. This also facilitates the upregulation of anti-apoptotic proteins (Bcl-2, Survivin) and downregulation of pro-apoptotic proteins (Bax), collectively contributing to cartilage repair and osteochondral regeneration [105]. The mechanical stimulation of UCMSC-Exos upregulates the long non-coding RNA H19 (LncRNA H19) in the Exos. Exosomal LncRNA H19 promoted chondrocyte proliferation and matrix synthesis, which are essential for cartilage repair. UCMSC-Exos with high LncRNA H19 expression led to better cartilage defect repair, surface regularity, integration, and GAG deposition and also alleviated pain during the early stages of cartilage repair, as shown in Figure 3d [94]. UCMSC-Exos secreted LncRNA H19 significantly promotes chondrocytes migration and matrix secretion, and suppresses apoptosis and senescence. The exosomal LncRNA H19 acts as a competing endogenous RNA (ceRNA) to sponge miR-29b-3p, which in turn upregulates Fo_x_O_3_ expression in chondrocytes, thereby enhancing osteochondral activity and supporting cartilage repair both in vitro and in vivo [106]. In another study, hBMSC-Exos loaded with miR-29a, encapsulated into a silk fibroin–chitosan (SF-CS) scaffold, mimic the physiological structure of natural cartilage and bone. The Exos-miR-29a enhance chondrocyte proliferation and differentiation, as evidenced by increased expression of COL-II and ACAN. In a full-thickness cartilage defect rat model, the scaffold loaded with miR-29a-Exos facilitated integration with surrounding bone and cartilage tissues, promoting cartilage tissue regeneration and subchondral bone repair [107].

The 3D-printed HG scaffold incorporates hBMSC-Exos and decellularized ECM (dECM) derived from cartilage (DCM) and bone (DBM), effectively delivers bioactive cargo molecules (proteins, metabolites, and nucleic acids), and promotes differentiation of MSCs into chondrogenic and osteogenic lineages, mimicking the natural osteochondral matrix. Exos combined with the dECM enhanced the expression of cartilage-specific genes (e.g., ACAN, COL-II, SOX9) and bone-related genes (ALP, OCN, COL-I, RUNX2), thereby simultaneously promoting the formation of hyaline cartilage and subchondral bone, offering significant potential for clinical translation in osteochondral defect repair [108]. MSC-Exos enhance the synthesis of cartilage matrix components, such as sulfated GAG (s-GAG) and COL-II, without compromising the chondrocytic phenotype. Exosomal CD73 converts extracellular adenosine monophosphate (AMP) into adenosine, thereby interacting with adenosine receptors on chondrocytes, triggering pro-survival signalling pathways such as AKT and ERK phosphorylation, which are essential for promoting chondrocyte survival, proliferation, and migration [109]. For instance, the sustained release of MSC-Exos from the 3D-printed cartilage ECM/GelMA scaffold restores mitochondrial function in chondrocytes, improves energy metabolism, and reduces oxidative stress damage, which are hallmarks of osteoarthritis. Moreover, the ECM/GelMA/Exos scaffold demonstrated robust in vivo performance by facilitating the formation of hyaline-like cartilage in defect areas and also enhancing the expression of cartilage matrix proteins (COL-2A1) while reducing cartilage degradation markers (MMP13). It also promotes the generation of ossified tissue in subchondral bone, increasing BV and Tb.Th. Furthermore, the scaffold polarizes macrophages toward an M2 phenotype, which supports tissue repair and reduces inflammation [102]. For instance, BMSC-Exos encapsulation within the HG scaffold creates a chemotactic environment that recruits endogenous BMSCs to the defect site, enhances BMSC migration and internalization, and forms a regenerative niche. The HG ensures the controlled release of Exos, protects their bioactivity, and promotes the differentiation of BMSCs into chondrocytes by upregulating SOX9, COL-II, and ACAN. In an animal OCD model, the HG accelerates hyaline-like cartilage regeneration, improves structural integration, and restores biomechanical properties [110]. Similarly, M2-Exos reduce pro-inflammatory cytokines such as iNOS, TNF-α, IL-1β, and CD86 in synovial tissue and increase anti-inflammatory markers such as ARG, PPARγ, CD163, and CD206. M2-Exos incorporated into ACECM scaffolds enhance cartilage repair specifically in a rat OCD model, as represented in Figure 3e [95]. For instance, icariin (ICA) encapsulated in BMSC-Exos improves the cellular uptake of ICA, which induces chondrogenic differentiation, enhances chondrocyte proliferation, reduces MMP13 secretion, and increases ECM production, while BMSC-Exos further amplify these effects by delivering ICA efficiently to cells and providing additional anti-inflammatory properties for OA treatment and cartilage protection [111].

WNT3a, a signalling molecule known to activate the WNT-β-catenin pathway, was loaded into conditioned medium-cultured L cell-derived Exos as a novel method for enhancing cartilage repair in osteochondral defects. These Exos serve as efficient carriers to deliver WNT3a into the dense, avascular cartilage matrix, thereby activating the WNT-β-catenin pathway in chondrocytes. The osteochondral defects created in the knee joint of an in vivo mouse model treated with WNT3a-L-Exos showed improved osteochondral repair compared to controls [112]. However, excessive activation and suppression of Wnt/β-catenin signalling can negatively impact cartilage health and contribute to OA, emphasizing the importance of maintaining balanced Wnt/β-catenin activity for joint health [113]. A recent study reported that the skeletal stem cell-derived Exos (SSC-Exos) loaded in the 3D-HG scaffold significantly enhances the chondrogenic differentiation of BMSCs through the delivery of miR-214-3p, which downregulates the jagged canonical Notch ligand 2 (JAG2), leading to the suppression of Notch signalling, thereby enhancing the chondrogenic differentiation of BMSCs and promoting cartilage repair. Moreover, in a rat OCD model, SSC-Exos-loaded scaffolds facilitated the simultaneous regeneration of articular cartilage and subchondral bone [114]. A recent study revealed that the hWJMSC-Exos stimulate the proliferation of BMSCs and chondrocytes, and induce macrophage polarization, which reduces inflammation in the joint cavity. The exosomal miRNAs, such as miR148a and miR29b, promote the synthesis of COL-II and proteoglycans, essential components of hyaline cartilage. Further incorporation of hWJMSC-Exos in the acellular cartilage extracellular matrix (ACECM) scaffolds shows enhanced osteochondral regeneration with improved structural and mechanical properties, including a higher Young’s modulus, similar to normal cartilage in the in vivo OCD rabbit model [103]. Contrastingly, a recent report demonstrated that hWJ-MSCs alone showed a better therapeutic effect in treating articular cartilage defects in a rat model than hWJMSC-Exos and their combination with HA. However, future research is strongly suggested to optimize the long-term outcomes for effective cartilage repair strategies [115]. In another study, human embryonic mesenchymal stem cell-derived Exos (hEMSC-Exos) were used to treat OCD in a rat model, resulting in hyaline cartilage formation, good surface regularity, and complete subchondral bone regeneration with no adverse inflammatory responses [116]. Moreover, the in vitro and in vivo mechanistic pathway analysis of Exos for bone and OCD regeneration is highlighted in Table 2.

## 5. The Current Progress in EV-Based Clinical Trials

Clinical translation of Exos and EV-based therapies for musculoskeletal disorders is currently emerging. Several registered early-phase studies focused on knee OA and investigated intra-articular (IA) delivery of MSC-derived EVs. Three ongoing phase I trials (NCT05060107; NCT06431152; NCT06466850) evaluate allogeneic MSC-EVs and UCMSC-EVs with dose escalation to establish safety and tolerability, and to assess improvements in pain and function. In another report, the clinical trial NCT04223622 used ADSC-derived secretome to treat arthritic osteochondral explants. The EVs, a key component of the secretome, were applied ex vivo to reduce the pathological phenotype of explanted chondrocytes. The results showed that EVs play a crucial role in cell communication, modulate the immune response to reduce inflammation, and are believed to contribute significantly to the therapeutic effects of the ADSC secretome in OA. The EVs counteract chondrocyte hypertrophy and inhibit the expression of catabolic factors. They contain factors such as TIMPs (Tissue Inhibitors of Metalloproteinases), DKK-1, and HGF, which help protect cartilage and reduce matrix degradation. Both soluble factors and EVs in the ADSC secretome are likely responsible for its beneficial effects, including anti-inflammatory and cartilage-protective properties [127]. For instance, a randomized triple-blind RCT of placental MSC-EVs (IRCT20210423051054N1) reported acceptable safety but no significant superiority versus a placebo following a single IA dose [128]. Additional registered studies include a U.S.-listed OA EV product (NCT06937528) [129], and two China-registered UCMSC-Exos for OA trials (ChiCTR2200059351; ChiCTR2500110969), indicating increasing global commitment to musculoskeletal applications. Across these clinical studies, CMC and quality control emphasize GMP-compliant manufacturing, scalable purification (e.g., TFF/SEC), and MISEV-aligned identity and purity markers (CD9/CD63/CD81, TSG101/ALIX; calnexin-negative) [130], alongside release testing for sterility, particle attributes, and potency assays aligned to joint repair mechanisms (e.g., IL-1β-suppressed chondrocyte catabolism) [131,132]. Biodistribution considerations justify IA administration, supported by preclinical evidence of sustained retention within the joint and minimal systemic exposure. Regulatory pathways treat EVs therapies as biologics or ATMPs, necessitating IND/CTA submissions with full comparability, CQAs, and defined release criteria. Notably, no registered EVs clinical trials for fracture non-union or spinal fusion exist, highlighting a translational gap between robust preclinical results and clinical adoption in bone-healing indications [133]. Furthermore, the clinical trials on bone and cartilage repair are highlighted in Table 3.

## 6. Conclusions and Future Perspectives

In conclusion, Exos are naturally occurring membrane-bound vesicles that are essential for intercellular communication, as they transport proteins, coding RNAs, and non-coding RNAs. This review facilitates the clinical functions of Exos in bone formation, including improving structural parameters, promoting vascularisation, and modulating key signalling pathways vital for osteogenesis and angiogenesis. Despite the encouraging findings, additional research is warranted to optimize their use and fully comprehend the underlying mechanisms for effective clinical translation. Various sources of stem cell-derived Exos and their engineered cargoes have been effectively applied to the treatment of bone-, cartilage-, and osteoarthritis-related medical conditions, resulting in improved therapeutic outcomes for patients. The integration of bioactive molecules and proteins with MSC-Exos holds significant potential to alleviate inflammatory responses, enhance immunomodulatory properties, mitigate OA progression, and improve cartilage regeneration. Building on current research advancements, future studies may focus more on engineered Exos in 3D culture conditions, introduce various therapeutically essential proteins and miRNAs to broaden their multifunctional therapeutic potential, and gain a deeper understanding of the mechanistic aspects to unravel the therapeutic needs. Furthermore, high-throughput technology, combined with advancements in biomaterial-based 3D printing, may enhance the self-healing integrated HG systems, thereby strengthening the targeted and sustained release of Exos and incorporated functional molecules, and providing promising treatment options in future regenerative medicine.

## Figures and Tables

**Table 1:** Comparative table of various Exos delivery vehicles in musculoskeletal indications.

Exos Source	Delivery Vehicle	Tissue Targeting	Exos Release		Mechanical Matching	References
			In Vitro	In Vivo		
3D-BMSC-Exos.	DSS6-peptide-modified GelMA (3D-SExos/DGel).	A mouse model of cranial bone defects.	At pH 7.4, 85% were released over 15 days.	3D-Exos and 3D-SExo were released rapidly after 7 days, while for 3D-SExo/DGel, the sustained release exceeded 21 days.	The Young’s modulus of HG is comparatively low, indicating that 3D-SExos encapsulation did not affect the HG.	[24]
MSC-Exos	ALG-HA/MSC-Exos HG.	Model of rat cranial bone defects.	After 14 days, the HGs of ALG:HA (5%:2.5%) released 79.20 ± 2.64% of Exos, ALG:HA (5%:5%) released 59.4 ± 3.24% of Exos, and ALG:HA (2.5%:5%) HG released 41.81 ± 4.91% of Exos.	The ALG-HA/EXOs (50%) group showed the highest osteogenic and angiogenic effects, leading to improved regenerative outcomes.	The maximum Young’s modulus was observed for the ALG:HA (5%:2.5%) and further decreased with (5%:2.5%) concentration. Moreover, the increasing elastic modulus after gelation provided physical support for bone regeneration.	[27]
BMSC-Exos	Different densities of (GelMA-30 and GelMA-60) HG. GelMA/BMSCs-Exos.	Rabbit TBJ model.	By the seventh day, approximately 80% of the Exos had been released.	NA	After optimization over (GelMA-30 and GelMA-60) HG for tendon–bone healing, GelMA-60-HG with higher porosity was selected for better encapsulation and release of Exos.	[29]
SC-Exos	Ti6Al4V/SC-Exos scaffold	New Zealand white rabbits with cylindrical bone defects created in the lateral femoral epicondyle.	NA	NA	3D porous Ti6Al4V scaffolds effectively reduce the elastic modulus of the Ti6Al4V implants, allowing them to match the natural bone tissue’s elastic modulus and reduce stress shielding.	[36]
BMP2-Exos.	BMP2-Exos-GelMA-HG.	Mice cranial defect model.	NA	Osteogenic differentiation and new bone formation indicate the sustained delivery of BMP2-Exos over 4–8 weeks.	The storage modulus (G’) and loss modulus (G’’) remained relatively stable with increasing angular frequency, indicating that Exos encapsulation did not alter the HG mechanical behaviour.	[37]
ADSC-Exos.	nHA/CS/PLGA scaffolds.	Maxillofacial bone defects.	Burst release was observed at 3 days (82.53 ± 17.91 µg), and then slowed to 105.16 ± 10.00 µg and 110.39 ± 11.75 µg on days 6 and 9, respectively.	Progressive scaffold degradation and Exos release were observed over 12 weeks.	The slow degradation rate of nHA and the inadequate mechanical strength of CS were improved when combined with PLGA, resulting in enhanced biocompatibility and osteogenic bioactivity.	[53]
BMSC-Exos.	3D-printed cartilage ECM/GelMA scaffold.	Chondrocytes and macrophages.	The scaffold retained Exos for 14 days, with a retention rate of over 56%.	ECM/GelMA-HG significantly retained Exos for at least 7 days, attributed to its COL fibril D-spacing, which helped retain Exos within the HG.	The photo-crosslinked ECM/GelMA-HG exhibited proper mechanical properties, with a Young’s modulus of 33.24 ± 8.8 kPa, which is suitable for load-bearing applications and effective for OCD repair.	[102]
hWJMSC-Exos	ACECM scaffolds.	OCD rabbit model.	NA	NA	The Young’s modulus of repaired tissue was higher in the hWJMSC-Exos/ACECM scaffold-treated group at 3 months than in other groups, but still lower than that of normal cartilage. By 6 months, the Young’s modulus was comparable to that of normal cartilage.	[103]

**Table 2:** Various types of stem cell-derived Exos and their molecular mechanistic pathway in the repair of bone and OCD.

Sources of Exos	Cell Target	Mechanism Impact	Biomedical Application	References
Serum-free conditioned medium-cultured BMSC-Exos	MSCs and vascular endothelial cells	Promote vascularisation and osteogenesis by upregulating angiogenic genes such as VEGF, ANG1, and ANG2, and by stimulating OB differentiation in MSCs.	Promote effective bone regeneration.	[117]
hDPSCs-Exos		Upregulate osteogenic markers like RUNX2, osteocalcin (OCN), bone sialoprotein (BSP), ERK phosphorylation, and ECM secretion.	Critical-sized bone defect repair.	[118]
BMSC-Exos	PDLCs	Enhanced PDL cell migration and proliferation through adenosine receptor-mediated activation of AKT and ERK signalling pathways.	Cell-free treatment option for periodontal regeneration.	[119]
hDPSCs-Exos	Bone marrow-derived macrophages	Decrease levels of inflammatory cytokines, such as IL-1β, TNF-α, and IL-6, and downregulate the NF-κB and p38 MAPK signalling pathways.	Therapeutic periodontitis.	[120]
rBMSC-Exos	HUVECs	Enhanced proliferation, migration, and tube formation, upregulated VEGF and CD31, and restored mitochondrial function in HUVECs exposed to high glucose.	Effective bone regeneration in diabetic environments.	[121]
BMSC-Exos	Chondrocytes	Suppress apoptosis, downregulate the expression of MMP-3 and MMP-13, increase the expression of LC3-II/LC3-I and Beclin-1, and decrease (Drp1) expression.	Protect chondrocytes and promote cartilage regeneration.	[122]
CC-Exos	Cartilage progenitor cells	Enhanced the expression of COL-II and SOX-9, chondrocyte differentiation, and matrix maturation, and prevented hypertrophy.	Improve cartilage regeneration.	[123]
BMSC-Exos	RAW264.7 macrophages and chondrocytes	Promote chondrocyte migration, modulate macrophage M2 polarization, reduce inflammation, and protect chondrocytes.	Improved articular cartilage repair.	[124]
Cartilage stem/progenitor cell-derived Exos (CSPCs-Exos)	Rat primary chondrocytes	Regulate pathways involved in cell migration and spindle organization, and reduce TNF and IL-17 production to mitigate inflammation.	OA model cartilage tissue repair.	[125]
UCMSC-EVs	Chondrocytes	Upregulated SOX-9, COL-II, and AGN, and downregulated Col-I, which is associated with fibrocartilage formation and regulates oxidative stress.	Cartilage regeneration.	[126]

**Table 3:** Registered and ongoing EV clinical trials in musculoskeletal indications.

Phase	Indication	EV Source	Dosing and Route	Primary Endpoints	Current Status	References
RCT	Knee OA	Placental MSC-EVs	Single IA dose (≈5 mL @ 7 × 10^9^ particles/mL)	WOMAC, MRI, safety	Completed—no superiority vs. placebo (IRCT20210423051054N1)	[128]
I (listed)	Knee OA/Osteoarthrosis	EV product (unspecified)	IA injection	Safety, function	Listed (NCT06937528)	[129]
I	Knee OA	Allogenic BM-MSC sEVs	Single IA injection (10^9^–10^10^ particles/mL)	Safety, WOMAC, pain/function	Active—not recruiting (NCT05060107)	[134]
I	Knee OA	UCMSC-EVs (EXO-OA01)	Single IA injection	Safety, cartilage thickness (MRI)	Recruiting (NCT06431152)	[135]
I	Knee OA	Allogenic MSC-EVs	Single/few IA doses	Safety, pain/function	Recruiting (NCT06466850)	[136]
Preclinical	Cartilage Repair	MSC-EVs + PRP/fibrin scaffold	Local delivery to the defect site	Histologic repair score, integration	Translational phase (no patients)	[137]

## Data Availability

No new data were created or analyzed in this study. Data sharing is not applicable to this article.
